# Knowledge and Performance on Intravenous Medication Administration to Patients: A Cross‐Sectional Observational Study Among Nurses of Nepal

**DOI:** 10.1002/nop2.70056

**Published:** 2024-10-10

**Authors:** Jaya Dhungana, Subina Bajracharya, Nancy R. Reynolds

**Affiliations:** ^1^ School of Nursing Chitwan Medical College Bharatpur Nepal; ^2^ Global Health Research & Medical Interventions for Development (GLOHMED) Kathmandu Nepal; ^3^ Johns Hopkins School of Nursing Baltimore Maryland USA

## Abstract

**Aim:**

To evaluate knowledge and performance levels regarding intravenous (IV) medication administration to patients among nurses working in paediatric centers of Nepal.

**Design:**

A cross‐sectional descriptive observational study.

**Methods:**

Enumerative sampling method was used for participant selection from the four hospitals having paediatric units. Data were collected using knowledge survey and observational checklist on four phases of IV medication administration.

**Results:**

Of 115 nurses, 14 (12.2%) had adequate knowledge about IV medication administration whereas none had good level and only 20 nurses (17.4%) had fair level of performance. There was a weak negative correlation between knowledge and practice scores. Nurses working 8‐h shifts had better performance than those working 6‐h shifts; however, the recommended nurse–patient ratio was not maintained in 80% of observed shifts. The findings highlight the importance of upgrading nurses' knowledge and professional competencies on medication administration to improve the quality of patient care.

## 1 Introduction

Medication‐related errors occur frequently in healthcare settings, accounting for one of 131 outpatient deaths and one of 854 inpatient deaths annually (Kapaki 2018). In the United States alone, 7000–9000 deaths occur yearly due to medication errors (Tariq et al. 2020). Most of the medication errors occur during drug administration to the patient, but these can also occur during dose preparation and documentation (Tariq et al. 2020). The rate of in‐patient medication errors is between 4.8% and 5.3% (Kapaki 2018). Among all medication errors, the incidence of errors related to intravenous (IV) medication is high in many countries (Fekadu et al. 2017).

There is paucity of evidence on medication administration‐related errors in low‐ and middle‐income countries (LMICs), including Nepal, which affects the design of capacity building interventions for the nurses.

## 2 Background

Nurses spend up to 40% of their time administering medication to the patients (Wondmieneh et al. 2020). As with any nursing care, medication administration also requires knowledge, skills and judgement. All Registered Nurses must apply their knowledge and skills during each of the four phases of medication administration: prescription and transcription, dose preparation, dispensing and administration, and monitoring‐evaluation (Wondmieneh et al. 2020). However, high work load and burnout among nurses, interruptions and distraction, unscientific nurse–patient ratio, variation in patient contact duration and illegible medical prescriptions can cause medication errors (Keers et al. 2013; Bucknall et al. 2019; Wondmieneh et al. 2020; World Health Organization 2020).

The incidence of medication error is three times higher in the paediatric patient population as compared to the adult patients (Feyissa et al. 2020). Incorrect dosing is the most common medication error in paediatric care (Wu 2019). Even a small error can pose a great risk, due to variation in the child's body mass, body volume (total body surface area) and organ development which guides an appropriate dosing (American Academy of Pediatrics 2003; D'Errico et al. 2022). The variation in concentrations and formulations of the medication may also trigger dosing errors. Therefore, nurses' level of knowledge, mathematical skills and technical proficiency are crucial for accurate weight‐adjusted dose calculation (Blignaut et al. 2017; Tsegaye et al. 2020; Wondmieneh et al. 2020; Elonen et al. 2022). Nurses need to be extra careful about administration of the right drug to the right patient through right route and accurate documentation of the whole process (Hanson and Haddad 2020). Moreover, the maintenance of standard nurse‐to‐patient ratio in each medical unit may help reduce medication‐associated errors and improve overall patient outcomes (Tubbs‐Cooley et al. 2013). The recommended nurse‐to‐patient ratio is 1:1 in the neonatal intensive care unit (NICU) and paediatric intensive care (PICU); 1:2 in Nursery; and 1:5 in general paediatric ward (Sharma and Rani 2020).

There are several studies conducted globally that assessed the knowledge and performance levels of nurses regarding medication administration; however, a very few studies have been conducted in the context of Nepal.

### 2.1 Aim

**FIGURE 1:**
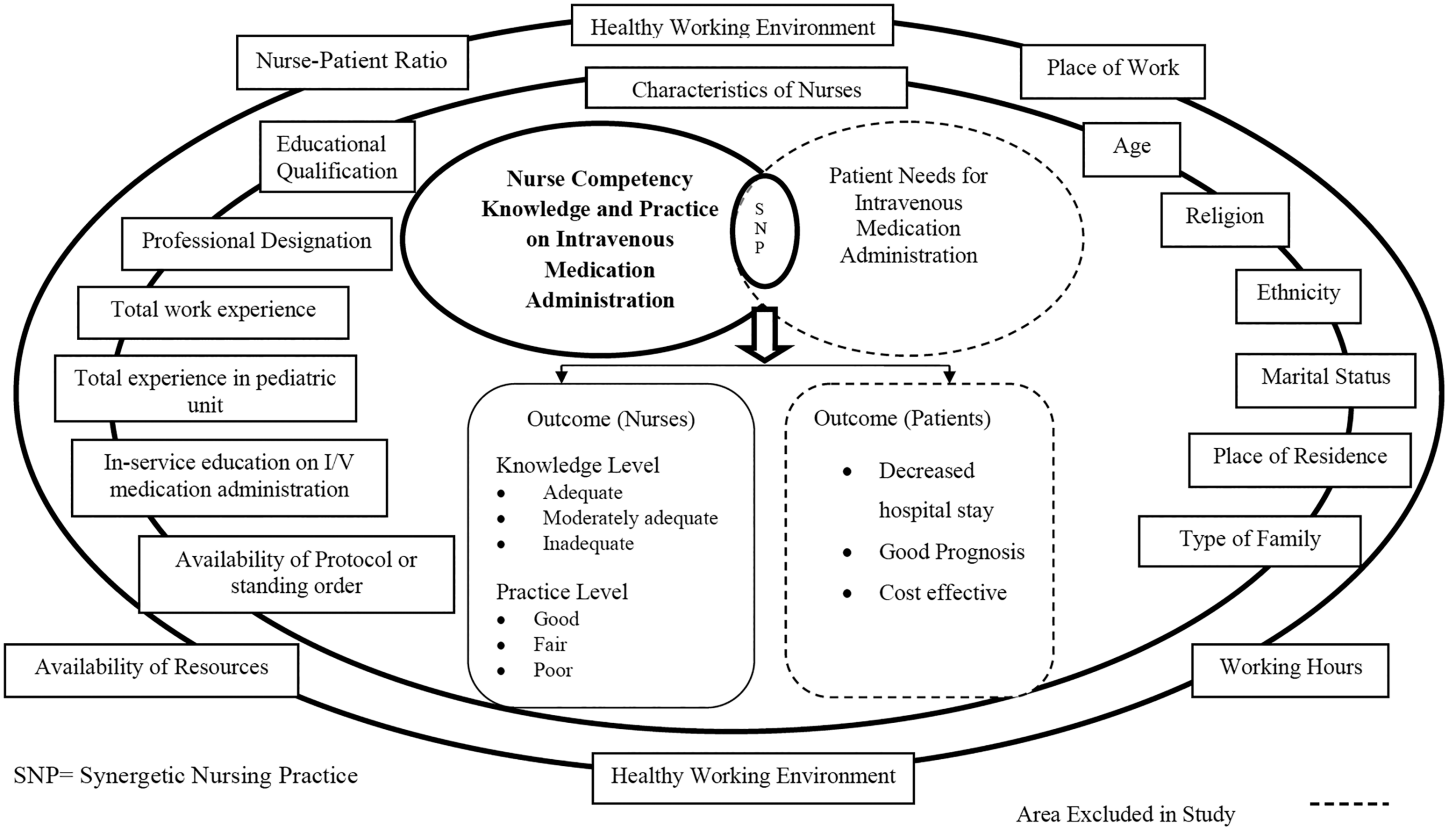
Conceptual model for nurses' knowledge and practice on IV medication administration adapted from Curley's patient–nurse synergy model (Curley 1998).

This study aimed to generate baseline evidence on nurses' knowledge and performance regarding IV medication administration to paediatric patients as well as the factors affecting their performance. The concept of the study was based on the synergy model (Figure 1), which emphasises the importance of alignment between patient needs and nursing competencies in achieving optimal patient outcomes and professional satisfaction (Curley 1998).

## 3 The Study

### 3.1 Design and Setting

A cross‐sectional descriptive observational study was conducted between August and September 2018 in the four hospitals located in Bharatpur Metropolitan City. The three hospitals were privately owned whereas one was public, and all four had paediatric care units. The study was conducted in the NICU, PICU, nursery and general paediatric ward of each hospital.

### 3.2 Participants and Sampling Technique

All nurses working in the NICU, PICU, nursery and general paediatric ward of the four hospitals were eligible for the study, except the head nurse of each unit who was pre‐informed about the study. A non‐probability total enumerative sampling method was used for data collection. Similar sampling method has been used by other studies (Pradhan et al. 2019; Koirala et al. 2019).

### 3.3 Study Tool

A study questionnaire was developed to assess the nurses' knowledge across four phases of medication administration, which were further divided into 18 items including dose calculation, documentation and patient rights. Likewise, nurses' performance was measured with a structured observational checklist developed based on the international standard nursing manuals for medication procedures and included 39 items such as medication preparation, history taking, administration techniques, patient communication, documentation, patient monitoring and patient evaluation (Japan International Cooperation Agency 2008; Nurses Association of New Brunswick 2016; New Zealand Nurses Organisation 2012). Content validity of the study tools was established by reviewing relevant literature and consultation with subject experts including members of the Nursing Research Thesis Committee, Tribhuvan University. Additionally, for the reliability of study tools, pretesting was conducted among 12 nurses (10% of total nurses) working in the paediatric unit of a non‐participating institution (Nepal Polytechnique Institute Narayani Samudayik Hospital and Research Center/NPI‐NSHRC) in the same setting. Furthermore, quantitative item analysis of the knowledge questionnaire was done for internal consistency (calculating the difficulty and discrimination indices) and tool modification.

### 3.4 Data Collection

The lead researcher collected data from the four study sites, recruiting four to five nurses from morning and evening duty shifts. Participant's knowledge and practice competence were measured across four phases of medication administration: *Phase 1* (*preparation and transcription*), *Phase 2* (*preparation*), *Phase 3* (*dispensing and administration*) and *Phase 4* (*monitoring and evaluation*). The self‐administered questionnaire was used to collect responses regarding the knowledge, whereas the lead researcher observed nurses for approximately 10 min and the performance of each step or procedure of medication was marked against the checklist. Each correct response or performance was scored ‘one’ and analysed accordingly.

### 3.5 Analysis

Nurses' knowledge level was categorised as: inadequate (≤ 50% of total score), moderately adequate (51%–74% of total score) and adequate (≥ 75% of total score; Ammu, Kumar, and Bashetti 2018). Similarly, the practice level was categorised as: poor (< 59% of total score); fair (59%–79% of total score); good (≥ 80% of total score) (Fashafsheh et al. 2015). The cut‐off points were decided after reviewing the literature that also suggested different terminologies for knowledge and practice (Ammu, Kumar, and Bashetti 2018; Fashafsheh et al. 2015).

Data were reviewed and checked for completeness and accuracy. After removing all personal identifiers, the coded data were entered in EpiData 3.1 version and then exported to the Statistical Package for Social Science (SPSS) 16.0 version. Descriptive statistics were used to estimate frequency, percentage, mean with standard deviation and mean of percentages. Inferential statistics such as chi‐square test, Yate's correction and Fischer's exact test were used to evaluate the association between level of knowledge or practice and selected variables. *p*‐value of ≤ 0.05 was considered statistically significant. Spearman's rank correlation coefficient (*r* = −1 to +1) was used to examine the relationship between knowledge and practice scores of nurses.

### 3.6 Ethics

Ethical approval was obtained from the Institutional Review Committee of Chitwan Medical College, Bharatpur, Nepal (Ref: CMC‐IRC/PG/075/076‐117). Additional approvals were received from the hospital administration as well as the Head of Nursing Department in each study site. A written informed consent was obtained from each study participant for knowledge assessment. Regarding performance assessment, a partially covert participant observation approach was followed, in which a written informed consent was obtained from the head nurse in each study site informing them of the research process (Roulet et al. 2017). Confidentiality of the participants was maintained throughout the study.

## 4 Results

### 4.1 Nurses' Characteristics and Work Scenario

Of 115 nurses who participated in the study, the majority (108, 93.9%) were between 19 and 29 years age (range 19–40 years) with the median age of 22 years. Only 17.4% of nurses (*n* = 20) had worked in the paediatric unit for more than 2 years. The majority (107, 93%) had obtained certificate level nursing education and only eight nurses (6.9%) had bachelors level qualification. Only 16.5% of nurses (*n* = 19) had received on‐job education or training specific to medication administration to the patients. Half of the nurses worked 6‐h shifts whereas the remaining worked 8‐h shifts. The recommended nurse‐to‐patient ratio was not maintained in the majority (91, 79.1%) of the shifts observed.

### 4.2 Nurses' Knowledge and Performance on IV Medication

Overall, only 14 (12.2%) nurses had adequate, 73 (63.5%) had moderately adequate and 28 (24.3%) had inadequate knowledge level on IV medication administration (Table 1). On practice domain, the majority (95, 82.6%) of nurses were found to be underperforming. Nurses' knowledge score was higher in Phase 3, that is, dispensing and administration (mean percentage: 72%) compared to Phase 1, that is, prescription and transcription phase (mean percentage: 50.66%). In contrast, nurses' practice score was comparatively higher in Phase 2, that is, preparation (mean percentage: 52.4%) than in other phases. The mean scores, as well as the mean percentages, of nurses' knowledge and practice for four phases of IV medication have been presented in Table 2. There was a weak negative correlation between nurses' knowledge and practice scores (*r* = −0.202, *p* = 0.031), suggesting that the higher level of knowledge regarding IV medication did not necessarily guarantee good practice.

**TABLE 1:** Nurses' knowledge and practice levels on IV medication administration (*N* = 115).

Level of knowledge	Frequency (%)	Level of practice	Frequency (%)
Adequate (≥ 75%)	14 (12.2)	Good (≥ 80%)	—
Moderately adequate (51%–74%)	73 (63.5)	Fair (59% to 79%)	20 (17.4)
Inadequate (≤ 50%)	28 (24.3)	Poor (< 59%)	95 (82.6)
Total	115	Total	115

**TABLE 2:** Nurses' knowledge and practice scores on four phases of IV medication administration (*N* = 115).

Phases of IV medication	Mean score ± SD (Mean %)	
	Knowledge	Practice
Prescription and transcription	1.52 ± 0.72 (50.66)	1.00 ± 0.13 (50.00)
Preparation	6.19 ± 1.62 (61.90)	13.10 ± 3.84 (52.40)
Dispensing and administration	2.16 ± 0.89 (72.00)	3.62 ± 1.86 (36.20)
Monitoring and evaluation	1.06 ± 0.76 (53.00)	0.23 ± 0.42 (11.50)
Total	10.93 ± 3.99 (60.72%)	17.95 ± 6.25 (46.02%)

#### 4.2.1 Knowledge About Four Phases of IV Medication

##### 4.2.1.1 Phase 1

Seven of every 10 respondents (80, 69.6%) correctly answered the chemical name of drug being administered.

##### 4.2.1.2 Phase 2

Nearly two‐thirds of respondents (73, 63.5%) answered the correct timing for hand hygiene, 95 (82.6%) correctly answered six rights of drug administration and n (48.7%) answered the right timing for three checks. Likewise, 92 (80%) knew the basis of drug dose calculation (either body weight or body surface area), 94 (81.7%) were aware about the angle at which vial is kept while drawing medicine and 108 (93.9%) answered correct dose calculation method.

##### 4.2.1.3 Phase 3

Majority (94, 81.7%) of the nurses correctly answered the actions required for cannula site redness and swelling, and 75.7% answered the correct handling of syringe following medication.

##### 4.2.1.4 Phase 4

Half (50.4%) of the nurses had knowledge on the method of measuring urinary output in infants following IV medication and 56.5% had knowledge regarding body site which needs to be monitored while administering intravenous potassium chloride.

#### 4.2.2 Performance on Four Phases of IV Medication

##### 4.2.2.1 Phase 1

Almost all nurses (114, 99.1%) verified the medication order using patient's chart, but only one nurse assessed the patient's history of drug allergies.

##### 4.2.2.2 Phase 2

Only 13 (11.3%) nurses washed their hands before administering IV medication and 40% checked for drug expiry date. Majority (93, 80.9%) prepared the patient's medication one at a time and almost all (113, 98.3%) verified the correct route of administration. While 84.3% correctly used appropriate syringe/needle size, only half of the nurses confirmed the right drug as per the prescription sheet. Only 60.9% (*n* = 70) could calculate the correct dose. Majority (104, 90.4%) checked the amount of drug in the syringe and discarded the surplus; 87% (*n* = 100) tapped the syringe to dislodge any air bubbles.

##### 4.2.2.3 Phase 3

High proportion of nurses (88, 76.5%) confirmed the right patient by checking the prescription sheet, but only 20 nurses (17.4%) verified it by checking the patient's identity card or calling out his/her name. Only one nurse informed the patient or attendant about the medication under use and its possible adverse effects. Very few nurses (19, 16.5%) checked the medication site for proper cannula placement, patency, leakage and phlebitis. While 72.2% of nurses (*n* = 83) properly administered the medication (slowly and gently for bolus and with calculated drop rate for infusion), 53.9% (*n* = 62) discarded the syringe in puncture‐proof container. Only 20 nurses (20.9%) washed their hands after medication administration.

##### 4.2.2.4 Phase 4

None of the nurses monitored the patient for immediate reaction or side effect of medication, whereas 27 nurses (23.5%) observed the patient for desired effects of medicine within 30 min of administration.

### 4.3 Factors Affecting nurses' Knowledge and Performance on IV Medication

Nurses' age, work experience, working unit, shift duration, on‐job training status, availability of medication protocol or standing orders and nurse‐to‐patient ratio were studied as potential factors affecting their knowledge and performance. When nurses' knowledge level was further re‐categorised into adequate (≥ 75% of total score) and inadequate (< 75% of total score), statistically significant association was found between knowledge level and working unit (Table 3). Nurses working in critical care units (NICU or PICU) had adequate knowledge than those working in general wards and nurseries (20.6% vs. 1.9%, *p* = 0.002). On performance domain, duration of shift was statistically significant; nurses' performance level was poor among those working > 6 h of work shift than those working 6‐h shift (96.6% vs. 68.4%, *p* ≤ 0.001). There was no significant association between nurses' knowledge or practice level and other factors (age, work experience, on‐job training status, availability of medication protocol or standing orders, nurse–patient ratio).

**TABLE 3:** Association of nurses' knowledge and practice levels with selected variables (*N* = 115).

Variables	Level of knowledge		*p*	Level of practice		*p*
	Adequate (%)	Inadequate (%)		Fair (%)	Poor (%)	
Age in years						
19–29	13 (12)	95 (88)	1.0^a^	20 (18.5)	88 (81.5)	—
30–40	1 (14.3)	6 (85.7)		0	7 (100)	
Working unit						
Critical care units	13 (20.6)	50 (79.4)	**0.002**	14 (22.2)	49 (77.8)	0.132
General paediatric ward and nursery	1 (1.9)	51 (98.1)		6 (11.5)	46 (88.5)	
Work experience						
≤ 2 years	10 (13.2)	66 (86.8)	0.881^a^	15 (19.7)	61 (80.3)	0.354
> 2 years	4 (10.3)	35 (89.7)		5 (12.8)	34 (87.2)	
Paediatric experience						
≤ 2 years	11 (11.6)	84 (88.4)	0.961^a^	17 (17.9)	78 (82.1)	1.0^a^
> 2 years	3 (15)	17 (85)		3 (15)	17 (85)	
Received on‐job education or training						
Yes	3 (15.8)	16 (84.2)	0.886^a^	3 (15.8)	16 (84.2)	1.0^a^
No	11 (11.5)	85 (88.5)		17 (17.7)	79 (82.3)	
Protocol or standing order available						
Yes	13 (12.3)	93 (87.7)	1.0^a^	20 (18.9)	86 (81.1)	—
No	1 (11.1)	8 (88.9)		0	9 (100)	
Nurse–patient ratio per shift						
Maintained	3 (12.5)	21 (87.5)	1.0^a^	2 (8.3)	22 (91.7)	0.311^a^
Not‐maintained	11 (12.1)	80 (87.9)		18 (19.8)	73 (80.2)	
Working hours						
6‐h shift	8 (13.8)	50 (86.2)	0.592	2 (3.4)	56 (96.6)	**< 0.001**
> 6‐h shift	6 (10.5)	51 (89.5)		18 (31.6)	39 (68.4)	

*Note:* Significant level at *p*‐value < 0.05, marked on bold.

^a^ Yates correction.

## 5 Discussion

The present study found that 12.2% of nurses working in the paediatric units had adequate, 63.5% had moderately adequate and 24.3% had inadequate knowledge level regarding IV medication administration to the patients. These findings are supported by a study conducted in India which reported 53.3% of nurses to have moderate and 46.7% inadequate knowledge (Samarasekera and Sathyadas 2016). The IV medication performance level was also poor in the majority (82.6%) of nurses. None of the nurses in our study were found to have a good performance level, as opposed to a study conducted in Kenya which reported 65% of nurses to have a good performance level (Kimeu 2015). There could be various reasons for lower knowledge and performance among nurses in our setting. First, the majority (93%) had received certificate level education, only eight nurses had completed bachelors level nursing education. Second, a high proportion of nurses were less experienced and there was a lack of on‐job education or training opportunities specific to medication administration. Arrangement of elective courses in specific practice areas such as medication procedures and medication calculations may enhance the knowledge and skills of nurses (Maneval et al. 2021; Elonen et al. 2022).

Medication error can occur due to mere procedural oversight, wrong dose calculation and wrong route of administration, as evidenced by studies conducted in Sweden and Ethiopia (Gunningberg et al. 2014; Tsegaye et al. 2020). Drug dose verification and expiry date check were not fairly practiced by the nurses in our study either, although the majority of them verified medicines in the prescription sheet and prepared one medication at a time. Nurses also poorly assessed the patients' history of drug allergy. Although the nurses in this study verified the correct route of drug administration, there are studies which report high number of medication errors due to drug administration via wrong route and wrong dose calculation (Tariq et al. 2020; Blignaut et al. 2017).

Nurse–patient ratio was not maintained in 80% of the shifts observed, which could be a factor for poor performance level of medication administration. The WHO Nursing Report 2020 as well as the facility‐based studies have reported nursing staff shortage as the major contributory factor to poor medication administration practice and hence high incidence of medication error and poor patient outcome globally (World Health Organization 2020; Kruk et al. 2018; Kimeu 2015; Salmasi et al. 2015; Tubbs‐Cooley et al. 2013).

This study did not find significant relationship between nurses' knowledge level and their age, work experience or training status. In contrast, studies conducted elsewhere found higher knowledge score among experienced or trained nurses than nurses with less experience or no training (Kerari and Innab 2021; Wondmieneh et al. 2020). Availability of medication protocol or standing orders may not guide adequate knowledge or good practice either (Tariq et al. 2020).

Our study further revealed that the nurses in low‐resource setting tend to neglect hand hygiene, a crucial preventive measure against infectious diseases. One of every five nurses did not wash hands before and after the medication procedure. It is important to increase nurses' compliance of hand hygiene to prevent patients from hospital‐acquired infections in paediatric settings. Nurses from Lebanon recommend the WHO‐5 multimodel intervention which includes staff education and training, system change, hospital reminders, direct observation and feedback, plus two additional measures, ownership of the initiative and goal setting, for the improvement of hand hygiene practices among health workers (Mrad et al. 2020).

The performance of nurses on IV medication was found relatively fair among nurses working 8‐h shifts compared to those working 6‐h shifts. Eight‐hour work shift is common in teaching hospitals of Nepal. In this regard, it is plausible to say that nurses working in teaching hospitals tend to have fair practice of IV medication compared to those working in non‐teaching hospitals. The nurses' medication performance, however, was not associated with their age, work experience and in‐service education or training, in contrast to the findings of a review that nurses' education level, experience and attendance at training courses are significantly associated with the occurrence and reporting of medication administration error (Kerari and Innab 2021; Asefa, Dagne, and Mekonnen 2021).

A weak negative correlation between IV medication knowledge and practice scores in this study suggests that higher knowledge level of nurses did not necessarily guarantee a good performance. This finding highlights the need to reform nursing education curricula to enhance the nurses' career readiness, for example, by allocating more teaching hours for hands‐on clinical teaching‐learning and arranging elective courses in specific identified specialties (Maneval et al. 2021). Formal clinical academic partnerships could be established between high‐ and low‐resource settings in order to foster continuing medical education programmes for nurses, fill in knowledge gaps in practices and improve patient outcomes (Baptiste, Whalen, and Goodwin 2022).

### 5.1 Strengths

The strengths of our study are mainly around its design and findings. All hospitals that provided paediatric care in the region were included (two of which were affiliated with medical schools) and all eligible paediatric nurses were given the opportunity to participate, so there was no question of sampling bias. Additionally, shift‐based observational technique was used to determine the difference between nurses' theoretical knowledge and its application; hence, it helped us understand the gaps in nursing care being delivered to the paediatric patients. Finally, our results can be good reference for policymakers as they prepare policies and guidelines on effective nursing care, with special consideration given to nurse–patient ratio per care unit, working hours per shift, in‐service training and provision of standing protocol.

### 5.2 Limitations

Our study has few limitations too. First, it assessed nurses' IV medication practice level during morning and evening work shifts only. It was not feasible to include night shifts despite the loose presumption that medication errors are more likely to occur during night hours. Second, the study was limited to the IV route of medication administration and did not include other forms of enteral and parenteral medication. Third, the study was one‐time cross‐sectional observation, but repeated observations would have been more helpful in estimating the nurses' knowledge and performance level on medication and factors associated with the low knowledge and performance. Some researchers suggest the role of automatic video monitoring of certain practices such as hand washing, drug preparation, and providing them with real‐time feedback for performance improvement (Mrad et al. 2020). Fourth, to avoid the Hawthorne Effect, all the participating nurses were kept unaware of the fact that their performance was being observed by the investigator. A partially covert participant observation approach was followed as the Head Nurses in each study site were pre‐informed about the observation and a written consent was obtained from them (Roulet et al. 2017). An ideal method of performance observation would be to take a written informed consent from the nurses themselves in advance for the deception, that is, informing them in advance that they were likely to be observed in the in‐patient paediatric units over the next few weeks while they provide care to the patients.

## 6 Conclusions

Nurses in the study setting had inadequate knowledge and poor performance on IV medication administration to paediatric patients. In‐service education and training including simulation exercises focusing on all the four phases of medication administration could help in upgrading nurses' knowledge and performance levels, and ultimately help in avoiding common medical errors. Moreover, further studies are required to know the exact factors associated with poor knowledge and skills in the nursing workforce. From the hospital management point of view, our study reinforces the importance of maintaining the standard nurse‐to‐patient ratio in each care unit so that nurses do not suffer from work‐related burnout and stress. From policy perspectives, all stakeholders of nursing education in a country which includes nursing division at the Ministry of Health, nursing council, nursing associations, medical education commission, national training centres and academic institutions should cooperate with each other for regular update of nursing curricula and provision of skill‐based in‐service education and training for nurses.

## Author Contributions

J.D. conceptualised the research, collected and analysed data and wrote the first draft of manuscript. S.B. supervised the research. S.B. and N.R.R. supported results interpretation and provided critical feedback to manuscript drafts. All authors reviewed and agreed to the submitted version.

## Ethics Statement

Ethical approval was obtained from the Institutional Review Committee (Ref: CMC‐IRC/PG/075/076‐117). A written informed consent was obtained from each study participant. Confidentiality was maintained throughout the study.

## Conflicts of Interest

The authors declare no conflicts of interest.

## Data Availability

The data that support the findings of this study are available from the corresponding author upon reasonable request.

## References

[nop270056-bib-0001] American Academy of Pediatrics . 2003. “Prevention of Medication Errors in the Pediatric Inpatient Setting.” Pediatrics 112: 431–436. 10.1542/peds.112.2.431. 12897304

[nop270056-bib-0002] Ammu, A. , S. S. Kumar , and S. Bashetti . 2018. “Assessment of the Level of Knowledge Regarding the Intramuscular Administration of Medication Among Staff Nurses.” Janaki Medical College Journal of Medical Science 5: 35–40. 10.3126/jmcjms.v5i2.19015.

[nop270056-bib-0003] Asefa, K. K. , D. Dagne , and W. N. Mekonnen . 2021. “Medication Administration Error Reporting and Associated Factors Among Nurses Working in Public Hospitals, Ethiopia: A Cross‐Sectional Study.” Nursing Research and Practice 2021: 1–8. 10.1155/2021/1384168. PMC8118739 34035959

[nop270056-bib-0004] Baptiste, D. L. , M. Whalen , and M. Goodwin . 2022. “Approaches for Establishing and Sustaining Clinical Academic Partnerships: A Discursive Review.” Journal of Clinical Nursing 31, no. 3–4: 329–334. 10.1111/jocn.15830. 33931906

[nop270056-bib-0005] Blignaut, A. J. , S. K. Coetzee , H. C. Klopper , and S. M. Ellis . 2017. “Medication Administration Errors and Related Deviations From Safe Practice: An Observational Study.” Journal of Clinical Nursing 26, no. 21–22: 3610–3623. 10.1111/jocn.13732. 28102918

[nop270056-bib-0006] Bucknall, T. , M. Fossum , A. M. Hutchinson , et al. 2019. “Nurses' Decision‐Making, Practices and Perceptions of Patient Involvement in Medication Administration in an Acute Hospital Setting.” Journal of Advanced Nursing 75, no. 6: 1316–1327. 10.1111/jan.13963. 30697809

[nop270056-bib-0007] Curley, M. A. Q. 1998. “Patient‐Nurse Synergy: Optimizing Patients' Outcomes.” American Journal of Critical Care 7: 64–72. 10.4037/ajcc1998.7.1.64. 9429685

[nop270056-bib-0008] D'Errico, S. , M. Zanon , D. Radaelli , et al. 2022. “Medication Errors in Pediatrics: Proposals to Improve the Quality and Safety of Care Through Clinical Risk Management.” Frontiers in Medicine 8: 814100. 10.3389/fmed.2021.814100. 35096903 PMC8795662

[nop270056-bib-0009] Elonen, I. , L. Salminen , I. Brasaitė‐Abromė , et al. 2022. “Medication Calculation Skills of Graduating Nursing Students Within European Context.” Journal of Clinical Nursing 31, no. 5–6: 548–558. 10.1111/jocn.15908. 34101280

[nop270056-bib-0010] Fashafsheh, I. , A. Ayed , F. Eqtait , and L. Harazneh . 2015. “Knowledge and Practice of Nursing Staff Towards Infection Control Measures in the Palestinian Hospitals.” Journal of Education and Practice 6, no. 4, Online, ISSN 2222–1735. https://files.eric.ed.gov/fulltext/EJ1083751.pdf.

[nop270056-bib-0011] Fekadu, T. , M. Teweldemedhin , E. Esrael , and S. W. Asgedom . 2017. “Prevalence of Intravenous Medication Administration Errors: A Cross‐Sectional Study.” Integrated Pharmacy Research and Practice 6: 47–51. 10.2147/iprp.s125085. 29354550 PMC5774322

[nop270056-bib-0012] Feyissa, D. , B. Kebede , A. Zewudie , and Y. Mamo . 2020. “Medication Error and Its Contributing Factors Among Pediatric Patients Diagnosed With Infectious Diseases Admitted to Jimma University Medical Center, Southwest Ethiopia: Prospective Observational Study.” Integrated Pharmacy Research and Practice 9: 147–153. 10.2147/iprp.s264941. 32983947 PMC7501953

[nop270056-bib-0013] Gunningberg, L. , U. Pöder , N. Donaldson , and C. Leo Swenne . 2014. “Medication Administration Accuracy: Using Clinical Observation and Review of Patient Records to Assess Safety and Guide Performance Improvement.” Journal of Evaluation in Clinical Practice 20: 411–416. 10.1111/jep.12150. 24798301

[nop270056-bib-0014] Hanson, A. , and L. M. Haddad . 2020. Nursing Rights of Medication Administration. Treasure Island, FL: StatsPearls Publishing. http://www.ncbi.nlm.nih.gov/pubmed/32809489. 32809489

[nop270056-bib-0015] Japan International Cooperation Agency . 2008. Fundamentals of Nursing Procedural Manual for PCL Course. https://www.jica.go.jp/nepal/english/office/topics/pdf/topics02_01.pdf.

[nop270056-bib-0016] Kapaki, V. 2018. “The Anatomy of Medication Errors.” In Vignettes in Patient Safety – Volume 4 [Working Title], edited by S. P. Stawicki and M. S. Firstenberg . London, UK: IntechOpen. 10.5772/intechopen.79778.

[nop270056-bib-0017] Keers, R. N. , S. D. Williams , J. Cooke , and D. M. Ashcroft . 2013. “Causes of Medication Administration Errors in Hospitals: A Systematic Review of Quantitative and Qualitative Evidence.” Drug Safety 36: 1045–1067. 10.1007/s40264-013-0090-2. 23975331 PMC3824584

[nop270056-bib-0018] Kerari, A. , and A. Innab . 2021. “The Influence of Nurses' Characteristics on Medication Administration Errors: An Integrative Review.” SAGE Open Nursing 7: 237796082110258. 10.1177/23779608211025802. PMC8223601 34222653

[nop270056-bib-0019] Kimeu, V. K. 2015. Factors Influencing Medication Administration Practice Among Nurses at Kenyatta National Hospital General Critical Care Unit. Nairobi, Kenya: University of Nairobi Research Archive. http://erepository.uonbi.ac.ke/handle/11295/94657.

[nop270056-bib-0020] Koirala, S. , R. K. Mehta , S. Acharya , and P. Gauro . 2019. “Critical Care Nurses' Views on Handover in Chitwan, Nepal.” In Connect: The World of Critical Care Nursing, vol. 13, 36–45. University of Alberta Library, Canada: International Journal of Critical Care. 10.1891/1748-6254.13.1.36.

[nop270056-bib-0021] Kruk, M. E. , A. D. Gage , C. Arsenault , et al. 2018. “High‐Quality Health Systems in the Sustainable Development Goals Era: Time for a Revolution.” Lancet Global Health 6: e1196–e1252. 10.1016/S2214-109X(18)30386-3. 30196093 PMC7734391

[nop270056-bib-0022] Maneval, R. , M. Hepburn , C. Brooks , et al. 2021. “Enhancing the Undergraduate Nursing Education Experience With Clinical Elective Courses.” Journal of Professional Nursing 37, no. 2: 366–372. 10.1016/j.profnurs.2020.04.014. 33867092

[nop270056-bib-0023] Mrad, Z. A. , N. Saliba , D. A. Merhi , A. Rahi , and M. Nabulsi . 2020. “Sustaining Compliance With Hand Hygiene When Resources Are Low: A Quality Improvement Report.” PLoS One 15, no. 11: e0241706. 10.1371/journal.pone.0241706. 33141855 PMC7608919

[nop270056-bib-0024] New Zealand Nurses Organisation . 2012. Guidelines for Nurses on the Administration of Medicines. https://edu.cdhb.health.nz/Hospitals‐Services/Health‐Professionals/IV‐Link/Documents/NZNOGuidelinesfornursesontheadministrationofmedicines.pdf.

[nop270056-bib-0025] Nurses Association of New Brunswick . 2016. Practice Standard Medication Administration. http://www.nanb.nb.ca/media/resource/NANB‐MedStandardRevised‐November2016‐E.pdf.

[nop270056-bib-0026] Pradhan, N. , S. Lama , G. Mandal , and E. Shrestha . 2019. “Physical Restraining: Nurses Knowledge and Practice in Tertiary Care Hospital of Eastern Nepal.” Nursing Open 6, no. 3: 1029–1037. 10.1002/nop2.298. 31367428 PMC6650657

[nop270056-bib-0027] Roulet, T. J. , M. J. Gill , S. Stenger , and D. J. Gill . 2017. “Reconsidering the Value of Covert Research: The Role of Ambiguous Consent in Participant Observation.” Organizational Research Methods 20, no. 3: 487–517. 10.1177/1094428117698745.

[nop270056-bib-0028] Salmasi, S. , T. M. Khan , Y. H. Hong , L. C. Ming , and T. W. Wong . 2015. “Medication Errors in the Southeast Asian Countries: A Systematic Review.” PLoS One 10: e0136545. 10.1371/journal.pone.0136545. 26340679 PMC4560405

[nop270056-bib-0029] Samarasekera, I. V. , and S. Sathyadas . 2016. “A Study To Assess the Knowledge Regarding Drug Dosage Calculation in Children Among Staff Nurses and Student Nurses in Narayana Medical College Hospital, Nellore.” Imperial Journal of Interdisciplinary Research (IJIR) 2, no. 4. Online, ISSN 2454‐1362. http://www.onlinejournal.in/IJIRV2I4/180.pdf.

[nop270056-bib-0030] Sharma, S. , and R. Rani . 2020. “Nurse‐To‐Patient Ratio and Nurse Staffing Norms for Hospitals in India: A Critical Analysis of National Benchmarks.” Journal of Family Medicine and Primary Care 9, no. 6: 2631–2637. 10.4103/jfmpc.jfmpc_248_20. PMC7491754 32984099

[nop270056-bib-0031] Tariq, R. A. , R. Vashisht , A. Sinha , and Y. Scherbak . 2020. Medication Dispensing Errors and Prevention. Treasure Island, FL: StatPearls Publishing. 30085607

[nop270056-bib-0032] Tsegaye, D. , G. Alem , Z. Tessema , and W. Alebachew . 2020. “Medication Administration Errors and Associated Factors Among Nurses.” International Journal of General Medicine 13: 1621–1632. 10.2147/IJGM.S289452. 33376387 PMC7764714

[nop270056-bib-0033] Tubbs‐Cooley, H. L. , J. P. Cimiotti , J. H. Silber , D. M. Sloane , and L. H. Aiken . 2013. “An Observational Study of Nurse Staffing Ratios and Hospital Readmission Among Children Admitted for Common Conditions.” BMJ Quality and Safety 22, no. 9: 735–742. 10.1136/bmjqs-2012-001610. PMC3756461 23657609

[nop270056-bib-0034] Wondmieneh, A. , W. Alemu , N. Tadele , and A. Demis . 2020. “Medication Administration Errors and Contributing Factors Among Nurses: A Cross Sectional Study in Tertiary Hospitals, Addis Ababa, Ethiopia.” BMC Nursing 19, no. 4. Online, ISSN 1472‐6955. 10.1186/s12912-020-0397-0. PMC6958590 31956293

[nop270056-bib-0035] World Health Organization . 2020. State of the World's Nursing 2020: Investing in Education, Jobs and Leadership. Geneva: World Health Organization. Online, ISBN 9789240003279. https://www.who.int/publications‐detail/nursing‐report‐2020.

[nop270056-bib-0036] Wu, A. 2019. Minimizing Medication Errors in Pediatric Patients. Virginia, USA: U.S. Pharmacist. https://www.uspharmacist.com/article/minimizing‐medication‐errors‐in‐pediatric‐patients.

